# Ensiling Process in Commercial Bales of Horticultural By-Products from Artichoke and Broccoli

**DOI:** 10.3390/ani10050831

**Published:** 2020-05-11

**Authors:** Paula Monllor, Gema Romero, Raquel Muelas, Carlos A. Sandoval-Castro, Esther Sendra, José Ramón Díaz

**Affiliations:** 1 Departamento de Tecnología Agroalimentaria, Universidad Miguel Hernández de Elche, 03312 Alicante, Spain; pmonllor@umh.es (P.M.); gemaromero@umh.es (G.R.); raquel.muelas@umh.es (R.M.); esther.sendra@umh.es (E.S.); 2 Facultad de Veterinaria, Medicina y Ciencia Animal, Universidad Autónoma de Yucatán, Mérida 97100, Mexico; carlos.sandoval@correo.uady.mx

**Keywords:** Silage, feedstuff, nutritive value, ruminant feeding, alternative feeds

## Abstract

**Simple Summary:**

Artichoke and broccoli crops are widespread throughout the world, mainly in the Mediterranean region. After artichoke harvests and industrial processing of artichoke and broccoli, large amounts of by-products are generated. The use of these alternative and cheaper feedstuffs in ruminants’ diets would reduce waste caused by the agri-food industry, disposal costs, and the land and natural resources used in animal feed production, contributing to the circular economy. Because of the high water content and the seasonality of these feedstuffs, ensiling might be a technology to preserve its nutritional quality for a long time, and this must be considered and studied at commercial scale. This paper looks into the viability of ensiling broccoli and artichoke by-products as commercial round bale silos (300 kg), their shelf life, and their suitability for ruminant feeding. The three silage by-products are stabilised on day 30. The high microbial quality and the appropriate nutritional composition at final stage (day 200) make them suitable for inclusion in ruminant diet, in combination with other energy and protein sources over a long period after the crop season.

**Abstract:**

Wastes from artichoke and broccoli crops and cannery industries represent an environmental problem. A viable option to this problem is ensiling them for use as ruminants feed. The aim of this study was to characterise the ensiling process of broccoli and artichoke by-products and assess their suitability to be part of the ruminant diet, as well their minimum shelf life. Twenty-one commercial round bale silos (300 kg and 0.64 m^3^) of each by-product were made. Samples were analysed at days 0, 7, 15, 30, 60, and 200 to determine microbial populations, fermentation metabolites, nutritional components, and phytosanitary residues. Feedstuffs showed good suitability for ensiling, and stabilisation was achieved on day 30. The variables with the greatest significant differences among sampling times were microbial populations and fermentative components. There were no important dry matter losses, and some significant differences were observed in the nutritional composition, especially in crude protein and fibrous fractions, but they were not relevant for the loss of nutritional quality of silages. The phytosanitary residues determined on day 200 were below the maximum residue limits set by European legislation. So, ensiling these by-products in commercial round bale silos is a suitable and profitable technique that allows their preservation for a long time (200 days).

## 1. Introduction

In 2017, 1,505,328 tons of artichoke (*Cynara scolymus* L.) and 25,984,758 tons of broccoli (*Brassica oleracea* var. *Italica*) were harvested worldwide [1]. Artichoke crops contribute significantly to the Mediterranean agricultural economy, the source of more than 60% of the world yield of this vegetable [2]. In the case of broccoli, more than 40% is produced in the Mediterranean and Southeast Asian regions [3]. 

The artichoke plant is a by-product consisting of leaves, stems and some inflorescences left in the field after harvest for human consumption. This stubble represents 80% of the total biomass of the plant [4] and has traditionally been used for feeding by grazing small ruminants or harvested and taken to farms [5]. Wernli and Thames [6] indicated that the yield was 11.105 t/ha of green forage in this crop, which, taking into account the FAO data on cultivated area worldwide (124,941 ha in 2016), would result in production figures of 1,387,470 t/year of available artichoke plant stubble. According to Ros et al. [7], 30% and 50% of the weight of processed broccoli and artichoke consist of by-products that could be destined for animal consumption and, considering the annual yield worldwide of these two vegetables, there will be 7,665,504 and 752,664 t of broccoli and artichoke by-products available, respectively.

Semi-intensive and intensive ruminant farms are characterised by high demand and dependence on feedstuffs, such as cereals, legume seeds, and alfalfa, which entail a high cost and require large crop areas. The use of local and alternative agri-food by-products and fodder in ruminant feeding reduces the external reliance on the aforementioned foods, thus minimising the use of land, water and supplies associated with livestock feeding and the feeding costs, which commonly exceed 60% of the total costs of a farm. Moreover, agricultural by-products, like artichoke and broccoli residues, are not competitive between human and livestock. Studies using other agricultural by-products have shown that if diets supplemented with this type of feedstuffs are formulated carefully, so that the nutritional needs of the animals are covered, their use may not compromise the milk yield and quality, or the technological properties and quality of the derived products [8]. Moreover, some recent publications showed that incorporation of by-products in diets would be profitable economically and nutritionally for dairy cattle production [9]. The use of broccoli and artichoke by-products can also be a way to minimise the waste originated by the canning industry and thus reduce disposal costs. However, the marked seasonality and high water content of these foods limit their direct use in animal feeding.

Ensiling is a way to preserve perishable products rich in lignocellulose and the main form of preservation of forage in many parts of the world, mainly due to its low dependence on climate, unlike hay [10]. Additionally, during ensiling, the bioactive components, such as polyphenols, undergo changes that might vary the antioxidant potential associated with them [11], which can positively affect animal health and performance, while improving the nutritional value of animal-derived products [12].

Only laboratory scale studies are published about artichoke and broccoli by-products silages [5,13,14,15], although no studies have been done in commercial size silos. The novelty of this experiment is that the evolution of these silos on a commercial scale are studied, so that the changes that occur during the silage process are better adjusted to reality, as they take place in a less controlled environment than laboratory scale studies. On the other hand, this study includes the variation of the microbiological populations of the three by-products, which better explains the changes in the fermentative and nutritional composition contents that occur in the three silages. Finally, no silage study with these by-products has reached the 200^th^ day, so this study provides additional information about silage quality over a longer period of time. For this reason, the results derived from this study provide a more realistic approach to the suitability of these by-products for ensiling. Several studies have shown that these by-product silages, in total or partial replacement of conventional forage sources, were a viable option for ruminant feeding [16,17,18,19,20,21,22,23,24].

The aim of this study is to characterise the silage process of broccoli and artichoke by-products and artichoke plant stubble in commercial size silos (300 kg round bale) over 200 days to determine the quality and suitability of these types of silages as a ruminant feed and its shelf life. Changes in variables related to the fermentation process, microbial population dynamics, nutritional composition, fermentative components, and in vitro digestibility were studied. The hypothesis of this study is that the commercial-scale silage technique of these by-products in 300 kg round-bale silos will allow food preservation for a long period of at least 200 days. The advantages of these types of silos over others are that they are easy to transport, can be sold as a package, have high compaction, good storage life, and no construction costs.

## 2. Materials and Methods 

**Figure 1:**
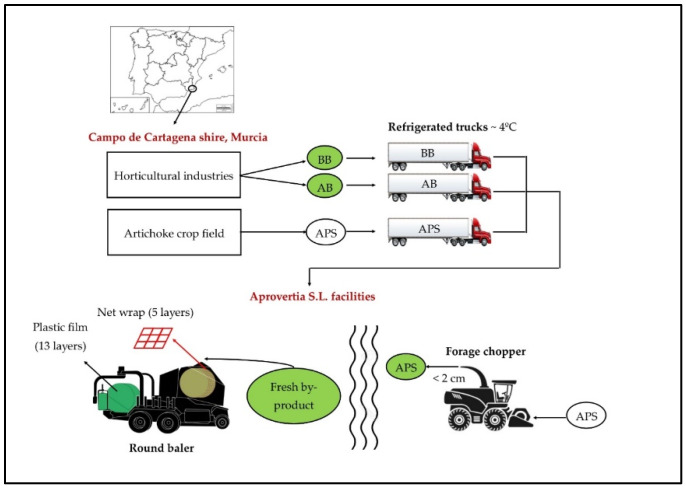
Ensiling process of the three by-products studied.

The materials studied were broccoli (*Brassica oleracea* var. *Italica*) and artichoke (*Cynara scolymus* L.) by-products (BB and AB, respectively) and artichoke plant stubble (APS). BB and AB were collected from agri-food industries from the Campo de Cartagena area, at the time they were leaving the horticultural facilities. APS was collected from a field of the same area after the artichoke harvest. All the materials were transported separately in refrigerated trucks to Aprovertia S.L. facilities, where the silo manufacturing took place. This process was in April, when the average temperature is 15 °C and relative humidity is 68% (values from San Javier, Murcia, Spain, weather station). To achieve suitable anaerobic conditions and correct compaction, the APS was cut and chopped to a size of 2 cm with a forage chopper (John Deere 8100, Moline, Illinois, USA) at silo manufacturing facilities. The same day, 21 round bale silos of each material of approximately 300 kg and 0.64 m^3^ each were made with a baler-wrapper (Agronic MR 820, Haapavesi, Finland), using five layers of inner conventional netwrap (Karatzis S.A., Heraklion, Greece) and 13 layers of conventional plastic film (Karatzis S.A., Heraklion, Greece) for each silo (Figure 1), following the description of Díaz et al. [25](2018) patent. No additives were used.

An experimental design was planned in which the sample collection days were 0 (silos manufacture day), 4, 7, 10, 15, 30, 60, and 200. For the sampling on day 0, three samples were collected from three different parts of the whole batch of the material to be ensiled. For the rest of the sampling days, each day a sample of 1 kg of three different bales was collected with a manual auger from three different zones of the bale: in the middle, and up, and down, 20 cm from the base. Then, the three sub-samples of a bale were mixed. Thus, three different representative samples of three different bales were obtained at every sampling day, which were taken to the laboratory, where they were separated into three aliquots: one for the analyses on the same day (dry matter, pH and microbiological cultures); another one was frozen at −20 °C; and another was dehydrated in an oven at 60 °C for 48 h.

On the same day of sampling, some variables were determined, such as pH (GLP 21, Crison, L’Hospitalet de Llobregat, Spain) and dry matter (DM, g/kg, AOAC, 1990, 948.12). With the samples from day 0, buffer capacity (meq NaOH 0.1N/100 g DM) was determined following the procedure in McDonald and Henderson [26]. Flieg scores were calculated to determine the quality of the silage, according to the equation given by Kilic [27]:

Flieg scores = 220 + (2 × DM (%) − 15) − 40 × pH(1)

According to this index, a score obtained below 20 would correspond to a very low quality silage; a low quality silage would have a score between 21 and 40; between 41 and 60 for medium quality silage; 61–80 in silages of high quality; and more than 81 for very high quality silages.

Samples for microbiological determinations were transferred to a laboratory in aseptic plastic bags. Then, 10 g of the sample was weighed into aseptic plastic bags with a lateral strainer and homogenised with 90 mL of peptone water in a stomacher (BagMixer^®^ 400, Interscience, St Nom la Bretèche, France). Microbiological cultures for counts of enterobacteria and aerobic mesophilic bacteria were directly incubated on 3M^TM^ Petrifilm plates (3M Microbiology, St. Paul, Minnesota, USA) at 37 °C for 24 h. Samples for lactic acid bacteria counts were incubated in MRS broth (Liofilchem, Roseto degli Abruzzi, Italy) at 37 °C for 48 h and moulds and yeasts were incubated on PDA (potato dextrose agar) culture, expressing the results as Log10 cfu/g of fresh matter and following AENOR procedures [28] in the fresh samples of days 0, 7, 15, 30, 60, and 200. 

Spore count of Clostridium genus was performed on days 0, 15 and 200, using the most probable number technique (MPN) and Bryant & Burkey broth (BBB, Merck, Darmstadt, Germany) following the methodology indicated in Arias et al. [29]. The microbiology cfu counts were transformed to log10 for further statistical analysis.

From samples dehydrated at 60 °C and further milled (1 mm), variables were analysed following AOAC procedures [30]. These variables were ash (g/kg DM, 934.01), crude protein (CP, g/kg DM, 988.05), ether extract (EE, g/kg DM, 920.39), crude fibre (CF, g/kg DM, 978.10), and total sugars (g/kg DM, 974.06). The contents of neutral detergent fibre (NDF, g/kg DM), acid detergent fibre (ADF, g/kg DM) and acid detergent lignin (ADL, g/kg DM) were also analysed according to Van Soest et al. [31]. In vitro dry matter digestibility (IVDMD, g/kg DM) was determined on days 0, 15, 30, 60, and 200, following the procedure of Menke and Steingass [32], using ruminal liquid extracted with oesophageal cannula from five goats from the farm of the Polytechnic School of Orihuela. A sample of 0.5 g was incubated in a 120 mL glass vial prewarmed at 39 °C before infusing with CO_2_ and the injection of 60 mL of buffer rumen liquor. The incubation was in an orbital bath at 39 °C. After 48 h, vial contents were filtered using Whatman paper no.2 and the residue was oven dried at 105 °C for 48 h. This study was approved by the Responsible Research Office of the Miguel Hernández University (code UMH.DTA.GRM.01.15). 

The frozen sample was used for analysis of the total polyphenols content (TP, g/kg DM) by the Folin-Ciocalteu method described in Kim et al. [33]. To study the fermentative changes during the ensiling process, some metabolites were measured, such as ammonia nitrogen (NH3-N, g/kg DM, 941.04) according to AOAC [30]; and short-chain volatile fatty acids (VFA, g/kg DM: acetic, butyric and propionic acids, although the latter was not found in the samples), lactic acid (g/kg DM) and ethanol (g/kg DM) by HPLC liquid chromatography (Agilent 1200, Santa Clara, California, USA and Supelcogel C-610H column: 30 cm × 7.8 mm ID, Saint Louis, Missouri, USA), as described by Feng-Xia et al. [34]. A multi-residual analysis of the main phytosanitary products used in artichoke and broccoli crops was carried out on the samples of day 200 using QuEChERS Method, according to Commission Regulation (EC) No 901/2009.

All the determined variables were analysed following a mixed linear model (Proc. GLIMMIX, SAS V 9.2, 2012) due to the heterogeneous nature of by-products, according to the following equation:

Y = μ + D_i_ + B_k_ + e (2)

Where Y is the dependent variable, μ is the intercept, D_i_ is the fixed effect of the ensiling day, B_k_ is the random effect of the bale, and e is the residual error. The covariance model with a lower value of the Akaike criterion (lower AIC and BIC) was used for each variable.

A calculation of the approximate costs of tested silos (€/t) was made, including raw material, inner netwrap and plastic film, workforce, and other production and marketing costs (the company’s commercial margin was not included).

## 3. Results

### 3.1. Microbiology

**Figure 2:**
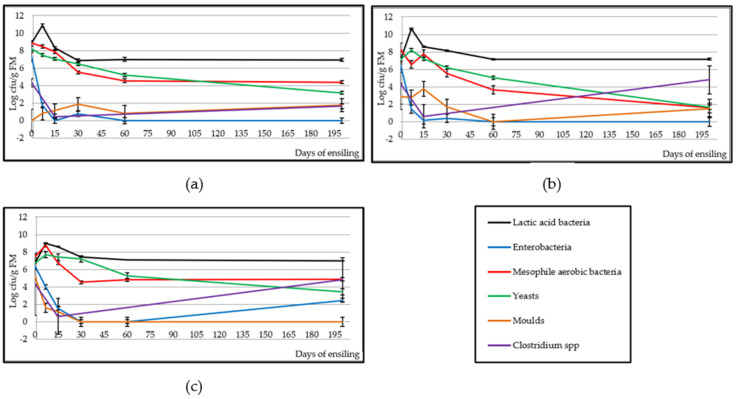
Effect of ensiling on microbial populations in broccoli by-product (**a**), artichoke by-product (**b**) and artichoke plant stubble (**c**) silages.

Figure 2 shows an increase in lactic populations (*p* < 0.001) in the early days (0–7) in the three silages, reaching the maximum on day 7 and becoming reduced and stabilised on day 30 in BB silage (6.88 log cfu/g FM) and on day 60 in AB and APS (7.13 and 7.08 log cfu/g FM, respectively). The enterobacteria population decreased markedly (*p* < 0.001) in the three silages from day 0 (7.26, 6.43 and 6.35 log cfu/g FM, respectively for BB, AB and APS) and disappeared completely on day 15 in BB and on day 60 in AB and APS, although in the latter there was a slight increase (*p* < 0.001) on day 200 (2.44 log cfu/g FM). The evolution of the mesophilic aerobes population varied according to the by-product. In BB, it decreased gradually (*p* < 0.001) from day 0 (8.84 log cfu/g FM) and stabilised on day 60 (4.56 log cfu/g FM), while in AB the reduction (*p* < 0.01) started on day 15 (7.77 log cfu/g FM) until day 200, reaching a value of 1.57 log cfu/g FM. In APS, a significant increase in mesophilic aerobes population was observed on day 7 (8.75 log cfu/g FM; *p* < 0.05)), followed by a reduction, reaching stabilisation on day 30 (4.52 log cfu/g FM). As for yeasts, it was not observed at any time that they stabilised, but their number in the three silages was reduced (*p* < 0.001) throughout the experiment, reaching 3.17, 1.65 and 3.42 log cfu/g FM on day 200, respectively for BB, AB and APS. Regarding moulds, in BB and AB they remained stable throughout the experiment, where the average value was 1.07 and 2.11 log cfu/g FM, respectively. However, in APS, a reduction (*p* < 0.001) was observed from day 0 (5.15) until moulds disappeared on day 30. The spore count of the genus *Clostridium* spp. reached its lowest (*p* < 0.05) value on day 15 in the three by-products and, subsequently, increased to levels similar to the initial ones, but without significant differences in AB and APS.

### 3.2. Physico-Chemical Parameters and Nutritional Composition

**Figure 3:**
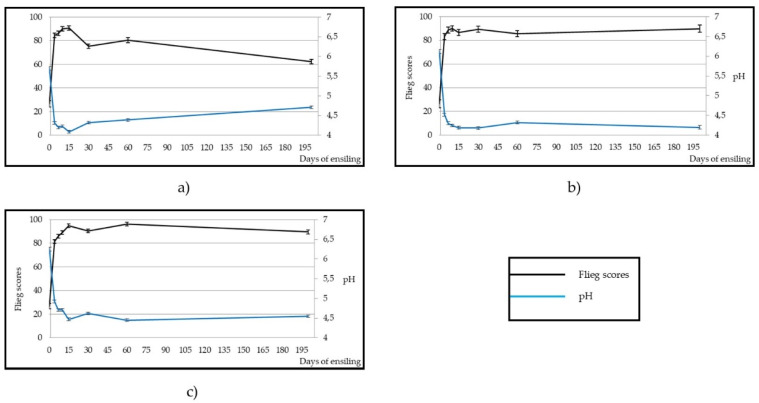
Effect of ensiling on Flieg scores and pH in broccoli by-product (**a**), artichoke by-product (**b**) and artichoke plant stubble (**c**) silages.

Figure 3 shows that pH decreased (*p* < 0.001) in the three silages, reaching its stabilisation on day 7 (4.30) in AB and on day 15 (4.47) in APS, while the lowest pH value of BB was observed on day 15 (4.09); thereafter, it increased slightly (*p* < 0.001) to reach a value of 4.71 on day 200. The buffer capacity of the raw materials before ensiling was 40.8, 21.8 and 19.6 meq/100 g DM in BB, AB and APS, respectively. Flieg scores increased to more than 80 from day 7 in all three silages, showing a very high quality. However, whereas AB and APS remained in this quality level, Flieg scores of BB decreased (*p* < 0.01) to the next lower level (60–80) from day 30, although silage quality was high quality.

Table 1 shows the changes in nutritional composition during the 200 days of silage. DM decreased (*p* < 0.001) in the three silages. While in AB and BB this decrease took place from the beginning until its stabilisation on day 30 (183 g/kg), in APS the value remained stable, decreasing slightly and gradually until reaching a value of 258 g/kg on day 200. While organic matter remained unchanged in AB during the whole experiment and its average value was 916 g/kg DM, it was slightly reduced (*p* < 0.001) in BB and APS, and reached values of 821 and 828 g/kg DM on day 200, respectively. Ether extract content increased slightly (*p* < 0.05) in BB and APS from day 30 and remained stable in AB. Crude protein level was very stable, except for a fluctuation (*p* < 0.001) observed in BB on day 60, in AB on day 200 and in APS on day 30. A decrease in NDF content was observed in the three silages from day 0 until day 30, then NDF increased on day 200 in BB and APS. Regarding ADF, AB did not show significant differences, BB and APS reduced their content until day 30 and 60, respectively, and then increased on day 200. The ADL content reduced from day 15 in BB and AB, and an increase (*p* < 0.05) on day 200 to levels similar to those on day 15 was observed in BB. In APS, the ADL level fluctuated slightly throughout the period, increasing on day 200. In vitro DM digestibility was reduced slightly (*p* < 0.01) in BB, while it increased in AB; in APS, it fluctuated with a few differences throughout the period. As for the content in TP, it rose (*p* < 0.001) in BB on day 15, remaining unchanged for the rest of the experiment; in AB it increased until day 60 and then decreased to a level similar to that presented on day 15, and in APS the value fluctuated slightly without major differences (*p* < 0.05).

### 3.3. Fermentation

**Figure 4:**
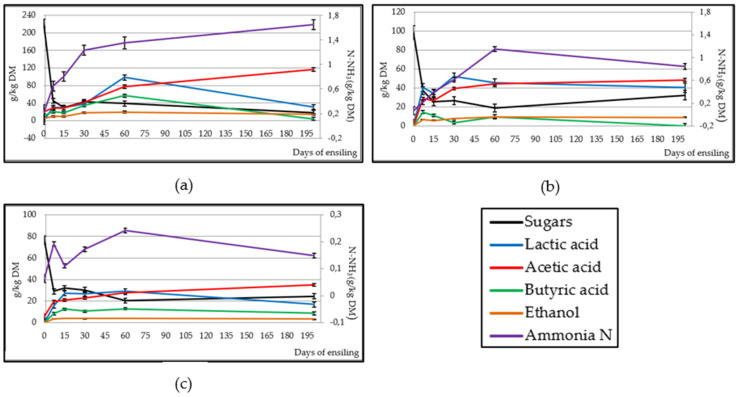
Effect of ensiling on sugar content and fermentative components in broccoli by-product (**a**), artichoke by-product (**b**) and artichoke plant stubble (**c**) silages.

Figure 4 shows a rapid reduction (*p* < 0.001) in sugar content in the threes silages from the outset, which ceased on day 15 in BB (29.2 g/kg DM) and AB (37.3 g/kg DM) and on day 7 in APS (29.0 g/kg DM). Lactic acid content increased (*p* < 0.001) in the three silages: while in BB and APS it reached its maximum value on day 60 (98.4 and 29.2 g/kg DM) and then was reduced (*p* < 0.001); in AB the maximum was observed on day 30 (51.9 g/kg DM) and remained at this level until the end of the experiment. Acetic acid concentration increased (*p* < 0.001) in the three silages gradually until the end of the experiment. Regarding butyric acid level, an increase (*p* < 0.001) was observed in the three silages: from day 30 in BB, where the maximum value was reached on day 60 (56.7 g/kg DM); the highest values in AB were given on days 7 and 15 (12.9 g/kg DM) and thereafter it was reduced (*p* < 0.001); in APS it increased until day 15 (12.6 g/kg DM), remaining stable until day 60. The ethanol concentration increased (*p* < 0.001) in the three silages from the beginning of the experiment, although it stabilised on different days: on day 30 for BB (17.8 g/kg DM), on day 60 for AB (9.19 g/kg DM) and on day 7 for APS (3.59 g/kg DM). As with the rest of the fermentation products, ammoniacal N increased in the three silages: It remained at a constant increase throughout the experiment in BB (*p* < 0.01) until day 200 (1.65 g/kg DM), while it reached its maximum value on day 60 (1.15 and 0.242 g/kg DM) in AB and APS, respectively.

### 3.4. Phytosanitary Residues Evaluation

Table 2 shows the phytosanitary residues determined on day 200 in the three silages. None of them exceeded the MRLs set by European legislation, although APS was the by-product in which a greater number of phytosanitary residues was detected.

### 3.5. Manufacturing Costs

Table 3 shows the different costs of the manufacturing process for the three types of silos. The main difference was observed in the cost of the raw material, since AB presented a higher cost (10 €/t of DM) than the other two by-products (4 and 5 €/tn of DM for BB and APS, respectively). However, final cost in €/kg of CP was higher in APS (1,01 €/kg CP) due to the lower content of CP of that silage.

## 4. Discussion

### 4.1. Microbiology

The lactic acid bacteria naturally present in silage are responsible for carrying out the fermentation and influence its final quality [35]. The same variation shown by the lactic acid bacteria population in this study was observed in Khota et al. and Wen et al. [36,37] in tropical grasses and alfalfa silages. The population of lactic acid bacteria remained above the other microorganism populations throughout the experiment. The number of colonies of the other microorganisms decreased as a result of the anaerobiosis conditions and the high level of lactic acid bacteria. According to Muller et al. [38], they inhibit the growth of harmful microorganisms due to the production of antimicrobial substances (bacteriocins). The enterobacteria population decreased more rapidly in BB and AB than in APS, because in the latter silage pH dropped more slowly due to a lower sugar content on day 0. Woolford [39] indicates that a pH lower than 4.5 reduces enzymatic activity and prevents the proliferation of enterobacteria. Enterobacteria count values are lower than those found in other studies [36,40,41], which are close to 4 Log10 cfu/g of FM from day 30 of ensiling. The yeast population was reduced due to the reduced availability of sugar and the increase in the concentration of acetic acid in the silages (Figure 4), which has antifungal and bactericide activity [42]. The low number of yeast colonies present after day 60 suggests that the silages will have high stability once the silo is opened, as the content was below the 6 Log10 cfu/g recommended by Kung et al. [43] and will have good acceptance by animals [44]. The mould population observed in the three silages is lower than that found by Junges et al. [45] in corn silages on day 90. The *Clostridium* spp. spores value was lower than those found by other authors in alfalfa silages (0.84 Log10 cfu/g on day 15, [37]) and corn silages (2–2.9 Log10 cfu/g on day 90, [45]).

Considering all the microbiological variables studied, it was shown that the concentrations of microorganisms potentially harmful to the silage quality were kept below levels taken as a reference from the works of other researchers, in the absence of a regulation that sets the maximum permitted limits for these microorganisms.

### 4.2. Physico-Chemical Parameters and Nutritional Composition

The main purpose of ensiling is to preserve the nutrients in fresh food with minimum losses of DM and energy. During ensiling, DM is lost as effluents and gases [46]. McDonald et al. [47] stated that the loss of DM due to the activity of lactic acid bacteria is between 2% and 5%, which is transformed into CO_2_ when the fermentation is carried out by heterofermentative bacteria. In this study, the loss of DM was between those values until day 30 in BB and day 60 in APS, while in AB a significant reduction in DM was observed from the beginning, although it subsequently stabilised. McDonald et al. [47] proposed 250 and 300 g/kg as appropriate initial DM contents for a correct silage process. Only in BB would it be below that range (174 g/kg), which would explain the slightest changes in the DM content in this silage, as happened in Megías et al. [14] with laboratory scale broccoli by-product silos. Wen et al. and Cai et al. [37,48] also found similar changes in DM content to those determined in this study in the final stage of silage. DM contents were higher in BB and AB silages of Megías et al. and Meneses et al. [13,14] (71.6 and 266 g/kg, respectively as mean values throughout the ensiling process), but DM of APS of this study was higher than Hernández et al. [5] (203 g/kg).

The buffer capacity of silages was lower than often found in other foods, such as tropical forages (67.11 meq/100 g DM) or corn (50.8 meq/100 g DM) [36]. This allowed the pH to drop more quickly in these silages than in those foods, decreasing the risk of nutrient loss and proliferation of undesirable microorganisms. The greatest pH drops occurred at the beginning of the experiment, coinciding with the increase in the lactic acid bacteria population, which resulted in a significant increase in the lactic acid content, which is the most effective acid to reduce the pH [14], and which serves as a good indicator of silage [49] quality. Although pH of AB started from a higher value compared to Meneses et al. [13] (6.12 vs. 5.84), the final pH value of both silages reached similar levels. According to Wen et al. and Kung et al. [37,42], the pH of alfalfa and grass silages with an initial DM of 250–380 g/kg should decrease to 4.2–4.7 to ensure silage quality. In this study, the starting points of DM in AB and APS were 256 and 283 g/kg, respectively, and the pH reached on day 200 was 4.20 and 4.55 for AB and APS. Additionally, the rate of lowering of the pH is as important as the final pH that is reached, as fast acidification lowers the risk of growth of undesirable microorganisms during the first stage of silage [50]. The greatest drop in pH happened during the first 7 days, concurrent with a peak of lactic acid bacteria populations and lactic acid increase on day 7 (Figure 2 and Figure 4), similarly to other studies [37,51,52]. 

Regarding Flieg scores, the values achieved were higher (indicating a higher quality) than those observed in other experiments with the same by-products, but with another ensiling technique, such as laboratory scale silos [14,18,21,53]. Only Meneses et al. [13] found a similar quality level for artichoke bracts laboratory scale silo on day 50 (95.6 Flieg points). However, in another experiment of the same researcher group with also laboratory size silos [54], the quality of artichoke bracts silage on day 100 was reduced to 64.2, whereas AB remained in the range 80–100 (very high quality) until day 200. 

BB composition values did not match those of broccoli by-products studied in Megías et al. [14] because those were different from the one used in this experiment, as they were boiled inflorescences and raw stems, showing the great variability in the composition of by-products of the same class, as indicated by García and Castrillo [55]. However, the composition of AB and APS were similar to that of Meneses et al. and Hernández et al. [5,13], as they were the same raw materials, although ensiled in laboratory conditions and with a shorter silage process. The reduction in protein content in BB and AB was not excessive, as it did not exceed the 15% established by Meneses et al. [13] as an indicator of excessive proteolysis due to slow fermentation [50]. However, this reduction was not observed in other studies with the same by-products, because in BB it increased and in AB it remained stable [13,14]. The phenolic compounds have strong antioxidant activity that affects the antioxidant potential of a feed [11]. The increase in TP agreed with the lactic acid bacteria-producing β-glucosidase that catalyses the release of the phenolics during ensiled fermentation, making them more accessible to the solvent during the extraction and, consequently, enhancing the silage antioxidative potential [56]. Jung [57] observed the same in alfalfa and corn silages: the increase in TP concentration reduced fermentation of structural carbohydrates. As shown in Table 1, higher values of TP were achieved from day 30, which coincides with the end of the reduction of the NDF content in the three silages studied. The greatest changes in NDF and ADF contents occurred in BB because this silage had a higher concentration of sugars on day 0; however, in this study, the fibrous fractions of BB were reduced, but in Megías et al. [14] they increased. Ashbell and Donahaye [58] indicated the relationship between the increase in ADF level as the sugar content is reduced, which causes an increase in the cell wall proportion in silage. Umana et al. [59] indicated that the highest digestibility of silage is achieved when populations of lactic acid bacteria stabilise, which also occurred on day 30 of this study. APS showed a higher IVDMD than Hernández et al. [5] in the same artichoke by-product (615 vs. 575 g/kg DM).

According to the silages composition on day 200, the high protein content and high DM digestibility of BB and AB increased CP/EM over 44 g of CP/Mcal EM (102 and 81.5 for BB and AB, respectively), which was proposed by García and Castrillo [55] as a good ratio for well-balanced feedstuffs. For this reason, these two silages (BB and AB) should be incorporated into the ration in combination with an energy source to make the diets meet the nutritional requirements for ruminants. Regarding APS, it showed a balanced CP/EM ratio (50.4 g of CP/Mcal EM), so the need for an energy source is less than with the other two silages.

### 4.3. Fermentation

The fermentation products strongly determine the hygienic and nutritional quality of the silage, affecting the voluntary intake and production of the animals and the composition and quality of the milk and its derivatives [8]. Consequently, the quantification of organic acids and alcohols resulting from fermentation is essential for evaluation of the silage quality [42]. Several factors influence the synthesis of these fermentation products, such as the predominant microorganisms in the silage, the fermentable substrates present, and the types of fermentation that take place during the whole silage process [47]. 

Lactic acid production rate depends on the initial lactic acid bacteria populations and the availability of an easily fermentable substrate, such as sugars [60]. The sugar decrease and lactic acid increase observed in this experiment also occurred in other silages [37,52]. The lactic acid concentration values obtained were lower than those often found in grass silage (60–100 g/kg DM), although similar to values in ensiled legumes (20–40 g/kg DM, [42]). Lactic acid content was lower in BB than in Megías et al. [14] (103 g/kg DM on day 50) because BB started from a lower sugar content (223 vs. 620 g/kg DM). Until day 60, the three silages kept the lactic acid level above the rest of the VFA and fermentation products, which indicates a good concentration of nutrients and good palatability and pleasant smell, which would lead to good acceptance and high consumption by animals [14]. However, on day 200, it was observed that the acetic acid content was higher than that of lactic acid in BB and APS, which is a consequence of prolonged fermentation [47] and would cause the loss of nutrients, as can be observed in the reduction in DM between day 60 and 200 for these two silages. AB showed a higher level of acetic acid than Meneses et al. [13] (48.3 vs. 12.5 g/kg DM), as well as in the rest of fermentation metabolites, as AB had a higher content of rapidly fermentable carbohydrates (sugars; 98.3 vs. 56.1 g/kg DM), which favoured a more intense fermentation. Although moderate concentrations of acetic acid in the ration can be beneficial, as it is absorbed in the rumen and used as energy or incorporated as fat into milk or body reserves [61], the lactic:acetic ratio is considered a good indicator of the type of fermentation that has occurred in the silage and its quality, the best levels being between 3 and 1 [42]. In this study, that ratio was achieved from day 7 until day 60, but the final acetic acid increase observed at day 200 caused a slight decrease in the ratio. The final value of acetic acid in BB was higher than that recommended by Kung et al. [42] in grass silage (10–30 g/kg DM). Butyric acid and ethanol levels remained low throughout the experiment because enterobacteria, clostridia and yeasts populations were reduced as a result of a high lactic acid bacteria population [47]. Ammonia N yield was higher in BB because of the extended fermentation process, which caused a slight proteolysis, but without great effects on the reduction of the nutritional content of the silage [13]. The fermentation product concentrations were similar to those found by Driheuis and van Wikselaar [62] in grass silage in which a lactic fermentation has taken place. 

The Flieg scale indicates that both AB and AP exhibited excellent silage quality. However, although BB quality remained high, it was on a lower level than the other two silages. Furthermore, its high CP content and high DM digestibility make it an interesting feedstuff to include in ruminant diets. Thus, the three silages can be used as part of livestock rations, although the high ammonia N concentration of BB may reduce its palatability and food intake in animals, so its inclusion in the diet should be done in moderation.

### 4.4. Phytosanitary Residues Evaluation

The phytosanitary residues found in the samples of the silages studied on day 200 do not exceed the MRLs imposed for each one by European legislation, so the three silages would not entail a risk to the health of the animals or for people who ingested the products obtained from these animals.

### 4.5. Manufacturing Costs

The cost of the three silages is much lower than that of other ingredients that are part of a conventional diet for ruminants. According to data prices from Lonja de Albacete [63] and feedstuff composition [64], alfalfa’s price would be 1.36 €/kg of CP and the price of cereals ranges from 1.63 €/kg of CP for barley to 1.87 €/kg of CP for wheat, so the minimum difference would be between alfalfa and APS (0.350 €/kg of CP) and the maximum between wheat and BB (1.16 €/kg of CP). For this reason, despite the silos manufacturing costs, the economic profitability of these by-product silages is very great.

## 5. Conclusions

According to the values obtained for microbiology, physico-chemical parameters and fermentative and nutritional components, stabilisation of studied by-products was achieved on day 30. Thereafter, most variables remained stable or were modified very slightly, as occurred with the count of microorganism populations. The silage’s quality remained high until day 200, as was the hypothesis of this experiment. We may state that ensiling broccoli and artichoke by-products and artichoke plant stubbles in commercial round bale silos is a practical and profitable technique that seems promising because it allows for their conservation over time, especially with artichoke by-products, not affecting their nutritional composition. Further studies should be carried out using them as feed for animals to explore voluntary intake and its effect on production and animal health.

## Figures and Tables

**Table 1:** Effect of ensiling on nutritional composition, in vitro dry matter digestibility and total polyphenols content in broccoli by-product (BB), artichoke by-product (AB) and artichoke plant stubble (APS) silages.

Silage	Days of Ensiling								SEM	*p*-Value
	0	4	7	10	15	30	60	200		
Dry matter (g/kg)										
BB	174bc	184ab	172bc	197ab	173bc	142d	181ab	154cd	6.79	***
AB	256a	220b	203bcd	201bcd	207bc	183d	192cd	190cd	8.66	***
APS	283abc	291abc	273cd	286abc	268d	277bcd	271cd	258e	4.21	***
Organic matter (g/kg DM)										
BB	849a				840a	826b	828b	821b	3.5	***
AB	912				927	912	912	916	9.4	n.s.
APS	839b				849a	837b	833bc	828c	2.3	***
Ether extract (g/kg DM)										
BB	20.2b				23.4b	29.1a	23.2b	32.1a	1.97	**
AB	19.6				29.3	25.8	28,0	29.6	3.41	n.s.
APS	26.3b				31.4ab	35.7a	34.7a	34.6a	2.22	*
Crude protein (g/kg DM)										
BB	195a				199a	204a	153c	174b	4.6	***
AB	117b				126b	125b	122b	145a	3.7	***
APS	78.7a				78.3a	67.1b	76.9a	78.1a	1.2	***
Neutral detergent fibre (g/kg DM)										
BB	395ab				356bc	311d	342cd	430a	11.7	***
AB	589a				530ab	510b	541ab	528ab	17.1	*
APS	547ab				555ab	540b	532b	571a	9.9	**
Acid detergent fibre (g/kg DM)										
BB	272b				266b	233c	259b	326a	8.2	***
AB	398				359	353	379	354	13.3	n.s.
APS	358ab				365a	361a	336b	374a	7.3	***
Acid detergent lignin (g/kg DM)										
BB	77.9a				65.0b	35.1c	35.1c	63.4b	3.63	***
AB	131a				81b	80b	84b	89b	6.8	***
APS	84.5bc				87.1b	91.9b	77.1c	107.9a	2.67	***
*In vitro* dry matter digestibility (g/kg DM)										
BB	888a				829b	857ab	800b	822b	14.7	**
AB	670b				724ab	723ab	737ab	769a	23.2	*
APS	606b				579c	632a	629ab	615ab	7.1	***
Total polyphenols (g/kg DM)										
BB	5.86c				8.85a	9.59a	7.48b	6.73ab	0.308	***
AB	1.96d				7.60c	10.08b	15.29a	7.56c	0.734	***
APS	5.02b				5.47ab	5.95a	5.55ab	4.96b	0.240	**

a–e Different letters in the same row indicate significant difference between days. * *p* < 0.05; ** *p* < 0.01; *** *p* < 0.001; n.s.: non significant.

**Table 2:** Phytosanitary residues (mg/kg) in silages after 200 days of ensiling in round bales broccoli by-product (BB), artichoke by-product (AB) and artichoke plant stubble (APS) silages.

Phytosanitary	Type	BB	AB	APS	MRL	Legislation
Cypermethrin	Insecticide	n.d.	n.d.	0.240	2.00	UE 520/2011
Chlorpyrifos	Insecticide	n.d.	n.d.	0.085	1.00	CE 839/2008
Imidacloprid	Insecticide	0.023	n.d.	0.034	0.50	UE 491/2014
Miclobutanil	Fungicide	n.d.	0.044	0.220	0.50	UE 2016/567
Spirotetramat	Insecticide	0.053	n.d.	n.d.	1.00	UE 2015/845
Triadimefon	Fungicide	n.d.	n.d.	0.700	1.00	CE 459/2010

MRL: Maximum Residue Limit; n.d.: Not Detected.

**Table 3:** Approximate manufacturing costs (€/t) of broccoli by-product (BB), artichoke by-product (AB) and artichoke plant stubble (APS) silages in 300 kg round bale silos on commercial scale.

Costs	BB	AB	APS
Raw material	4	10	5
Inner netwrap and plastic film	7.9	7.6	8.1
Workforce, other production and marketing costs	7.3	7.3	7.3
Total (fresh matter)	19.2	24.9	20.4
Total (dry matter)	125	131	79
Total (€/kg CP)	0.718	0.903	1.01

CP: crude protein.
